# Antitumor Activity of *Ruditapes philippinarum* Polysaccharides Through Mitochondrial Apoptosis in Cellular and Zebrafish Models

**DOI:** 10.3390/md23080304

**Published:** 2025-07-29

**Authors:** Mengyue Liu, Weixia Wang, Haoran Wang, Shuang Zhao, Dongli Yin, Haijun Zhang, Chunze Zou, Shengcan Zou, Jia Yu, Yuxi Wei

**Affiliations:** 1 College of Life Sciences, Qingdao University, Qingdao 266071, China; lmy02112021@163.com (M.L.); zhaoshuang2544@163.com (S.Z.); 2 Qingdao Yihai Industry Holdings Co., Ltd., Qingdao 266105, China; wangweixia@chenland.cn (W.W.); yindongli@chenland.cn (D.Y.); zhanghaijun@chenland.cn (H.Z.); zoushengcan@chenland.cn (S.Z.); 3 College of Materials Science and Engineering, Qingdao University, Qingdao 266071, China; 19817801579@163.com; 4 Donald Bren School of Information and Computer Sciences, University of California, Irvine, CA 92697, USA; chunzez@uci.edu

**Keywords:** *Ruditapes philippinarum* polysaccharides, anti-colorectal cancer activity, zebrafish, mitochondrial apoptosis

## Abstract

Colorectal cancer (CRC) remains a predominant cause of global cancer-related mortality, highlighting the pressing demand for innovative therapeutic strategies. Natural polysaccharides have emerged as promising candidates in cancer research due to their multifaceted anticancer mechanisms and tumor-suppressive potential across diverse malignancies. In this study, we enzymatically extracted a polysaccharide, named ERPP, from *Ruditapes philippinarum* and comprehensively evaluated its anti-colorectal cancer activity. We conducted in vitro assays, including CCK-8 proliferation, clonogenic survival, scratch wound healing, and Annexin V-FITC/PI apoptosis staining, and the results demonstrated that ERPP significantly inhibited HT-29 cell proliferation, suppressed colony formation, impaired migratory capacity, and induced apoptosis. JC-1 fluorescence assays provided further evidence of mitochondrial membrane potential (MMP) depolarization, as manifested by a substantial reduction in the red/green fluorescence ratio (from 10.87 to 0.35). These antitumor effects were further validated in vivo using a zebrafish HT-29 xenograft model. Furthermore, ERPP treatment significantly attenuated tumor angiogenesis and downregulated the expression of the vascular endothelial growth factor A (*Vegfaa*) gene in the zebrafish xenograft model. Mechanistic investigations revealed that ERPP primarily activated the mitochondrial apoptosis pathway. RT-qPCR analysis showed an upregulation of the pro-apoptotic gene *Bax* and a downregulation of the anti-apoptotic gene *Bcl-2*, leading to cytochrome c (*CYCS*) release and caspase-3 (*CASP-3*) activation. Additionally, ERPP exhibited potent antioxidant capacity, achieving an 80.2% hydroxyl radical scavenging rate at 4 mg/mL. ERPP also decreased reactive oxygen species (ROS) levels within the tumor cells, thereby augmenting anticancer efficacy through its antioxidant activity. Collectively, these findings provide mechanistic insights into the properties of ERPP, underscoring its potential as a functional food component or adjuvant therapy for colorectal cancer management.

## 1. Introduction

In recent decades, a steady increase in the incidence of colorectal cancer (CRC) among adults has been documented, predominantly linked to deleterious dietary patterns [1]. Surgical intervention, frequently combined with radiotherapy or chemotherapy, remains the primary therapeutic strategy for CRC. However, these conventional treatments are often associated with severe adverse effects [2]. In addition to surgical resection, various pharmacological agents are routinely employed in the clinical management of CRC. Commonly used agents such as oxaliplatin, irinotecan, and fluorouracil are associated with adverse effects, including diarrhea, nausea, vomiting, and hand–foot syndrome [3,4]. While immunotherapy has emerged as a promising alternative, its efficacy has been limited by heterogeneity in patient immune profiles, especially among different CRC molecular subtypes [5,6].

Natural anticancer agents, particularly bioactive polysaccharides, have drawn substantial scientific attention [7,8]. Lentinan (LNT), a fungal β-glucan, has been reported to enhance therapeutic responses in advanced gastric cancer [9]. Recently, the focus has shifted toward marine-derived polysaccharides, which exhibit unique structural features and diverse biological activities. Polysaccharides isolated from marine life have been investigated for their pharmacological properties, such as the anticoagulant activity in sea cucumber-derived fucosylated chondroitin sulfate [10], the antitumor effects of *oyster* (OP-1) and *abalone* (AVP) polysaccharides [11,12], and the antioxidant capacity of starfish-derived SF polysaccharide [13].

*Ruditapes philippinarum*, a commercially significant bivalve mollusk, has been extensively cultivated in the coastal regions of China, Korea, and Southeast Asia, accounting for approximately 90% of global production [14,15]. In addition to its nutritional value, this species has a long-standing history of use in traditional Chinese medicine due to its alleged anti-inflammatory and expectorant properties [16]. Previous investigations of microwave-extracted clam polysaccharides demonstrated significant in vitro antitumor efficacy and immunomodulatory activity [17]. However, their antitumor potential remained uncharacterized in vivo.

In recent years, the zebrafish has emerged as a powerful platform for modeling and investigating tumor suppressor gene function [18]. This utility is derived from its ease of genetic manipulation, unique advantages in live imaging, potential for high-throughput screening, and the evolutionary conservation of tumor biology with humans. The rapid increase in the number and expansion of the application scope of zebrafish tumor models has significantly enhanced our understanding of tumorigenesis mechanisms and accelerated the processes of anticancer drug discovery and translational research [19]. Previous investigations on *Rosa roxburghii* polysaccharide (RRTP80-1) have demonstrated a significant suppression of A549 xenograft tumor growth in zebrafish models [4].

This study systematically evaluated the antitumor activity and underlying mechanisms, both in vivo and in vitro, of polysaccharides extracted from *R. philippinarum* (ERPP) through enzymatic hydrolysis. The findings offer critical insights into the therapeutic potential of ERPP as a novel marine-derived agent for functional food or pharmaceutical applications in oncology.

## 2. Results

### 2.1. Antitumor Activity of ERPP In Vitro

#### 2.1.1. Effects of ERPP on Multiple Cancer Cell Lines

**Figure 1:**
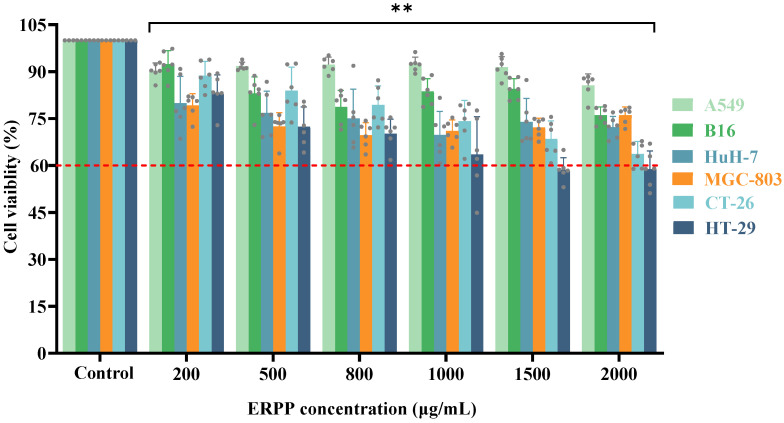
The anti-proliferative activity of ERPP was assessed in multiple cell lines. Values are the mean ± SE (*n* = 6). ** *p* < 0.01, compared to the control group.

As shown in Figure 1, the inhibitory effects of ERPP on multiple cancer cell lines (A549, B16, HuH-7, MGC-803, CT-26, and HT-29) were evaluated using CCK-8 assays [20]. Cells cultured in a polysaccharide-free medium served as the negative control. In all cell lines treated with ERPP, a dose-dependent growth inhibition was observed, with the most pronounced suppression detected in HT-29 cells. When exposed to the highest concentration of ERPP, the viability of HT-29 cells dropped below 60%. Based on these results, further antitumor activity investigations were specifically focused on HT-29 cells.

#### 2.1.2. Attenuation of Colony Formation and Migration Capacity in HT-29 Cells by ERPP

**Figure 2:**
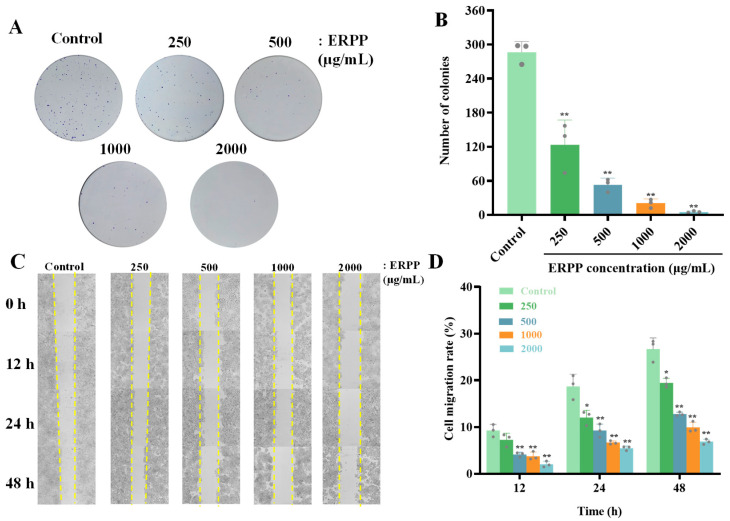
ERPP reduces cell colony generation and migration of HT-29 cells. Colony formation assay (**A**,**B**) and wound healing (**C**,**D**). Experiments were conducted using three independent biological replicates, and data of representative images are shown. Values are mean ± SE (*n* = 3). * *p* < 0.05 and ** *p* < 0.01 compared to control group.

The colony formation assay evaluates clonogenic survival through single-cell proliferative capacity, a key determinant of the in vitro tumorigenic potential, while the scratch assay quantifies treatment-induced alterations in cellular migratory capacity [21,22]. HT-29 colony formation was significantly suppressed by ERPP treatment compared to the control group in a dose-dependent manner (Figure 2A). The high-dose group exhibited a remarkable 97.2% reduction in colony formation (Figure 2B).

The in vitro scratch assay is a cost-effective and well-established method for assessing cell migration. It involves creating a confluent monolayer scratch, capturing time-lapse images during wound closure, and quantifying cellular migration rates through comparative analysis [23]. The effects of ERPP on wound healing were further investigated, demonstrating a significant inhibition of HT-29 cell migration (Figure 2C). In wound healing experiments, treatment with high-dose ERPP resulted in a significantly reduced migration rate (6.83%) versus the blank control (26.65%) (Figure 2D). Collectively, these findings demonstrate the substantial inhibitory effect of ERPP on HT-29 tumorigenesis.

#### 2.1.3. ERPP Induced Apoptosis in HT-29 Cells

**Figure 3:**
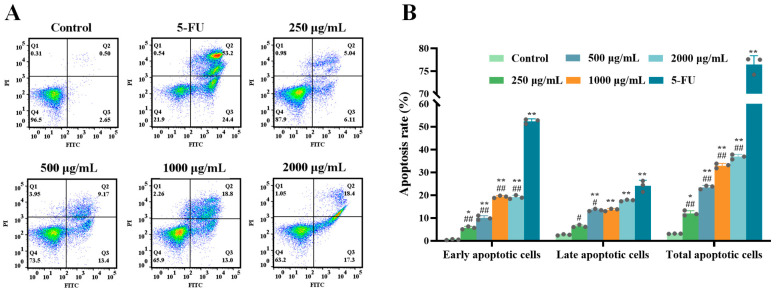
Effect of ERPP on apoptosis of HT-29 cells. Flow cytometry chart illustrating apoptotic degree (**A**) and bar chart illustrating percentage of apoptosis (**B**). Values are mean ± SE (*n* = 3). ** *p* < 0.01, * *p* < 0.05 compared to control group. ^##^ *p* < 0.01, ^#^ *p* < 0.05 compared to 5-FU group.

Previous research has indicated that *Ruditapes philippinarum* polysaccharides can trigger apoptosis in cancer cells, suggesting a potential apoptotic induction effect of ERPP on tumor cells [24]. Apoptosis in ERPP-treated HT-29 cells was quantified using Annexin V-FITC/PI dual staining followed by flow cytometric analysis. Flow cytometric analysis revealed significantly increased apoptotic cell populations in the Q2 (late apoptosis) and Q3 (viable cells) regions following ERPP and 5-FU treatment (Figure 3A). A dose-dependent increase in total apoptosis rates was observed in the ERPP-treated groups, rising from 3.04% (untreated control) to 36.81% at the highest concentration. The pro-apoptotic efficacy of ERPP reached 47.92% of that achieved in the 5-FU-treated positive control group. (Figure 3B). These experimental results demonstrate the efficacy of ERPP in inducing apoptosis in HT-29 cells.

#### 2.1.4. Effects of ERPP on Mitochondrial Membrane Potential (MMP)

The functional assessment of mitochondrial membrane potential (MMP) was conducted in HT-29 cells. The JC-1 fluorescent probe was employed to evaluate MMP alterations, as depolarization (indicated by reduced red/green fluorescence ratio) represents an early apoptotic event associated with mitochondrial permeability transition pore opening [25,26,27].

**Figure 4:**
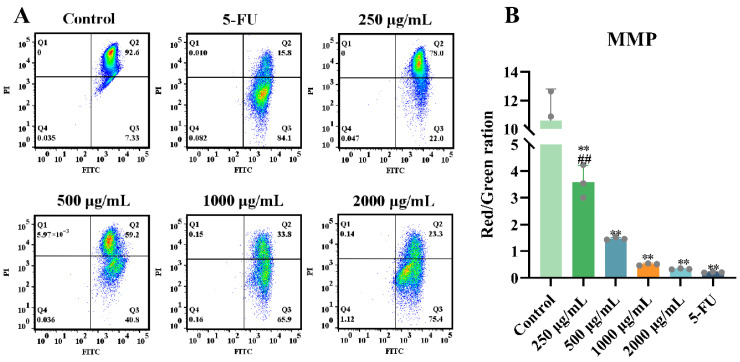
Effect of ERPP on mitochondrial apoptosis of HT-29 cells. Loss of mitochondrial membrane potential (MMP) was tested by JC-1 staining (**A**) and bar charts of MMP changes (**B**). Values are mean ± SE (*n* = 3). ** *p* < 0.01 compared to control group. ^##^ *p* < 0.01 compared to 5-FU group.

In ERPP-treated cells, a significant reduction in red fluorescence intensity and an increase in green fluorescence were observed (Figure 4A), indicating MMP collapse and enhanced mitochondrial membrane permeability. A concentration-dependent decrease in the red fluorescence percentage was detected in HT-29 cells, dropping from 92.6% to 23.3% following ERPP treatment. Concurrently, the red/green fluorescence ratio decreased from 10.87 to 0.35 (*p* < 0.01), suggesting mitochondrial membrane depolarization, which is a hallmark of apoptosis (Figure 4B). In the control groups, the mitochondria exhibited predominant red fluorescence, while ERPP-treated cells displayed a dose-dependent increase in green fluorescence intensity, further confirming MMP collapse. In conclusion, these results showed that the mitochondria are involved in ERPP-induced apoptosis.

### 2.2. Antitumor Activity of ERPP In Vivo

#### 2.2.1. Effects of ERPP on Tumor Proliferation in HT-29 Zebrafish Xenografts

To further evaluate the antitumor potential of ERPP in vivo, an HT-29 xenograft zebrafish model was utilized. The maximum tolerable concentration (MTC) of ERPP was determined to be 125 μg/mL under these experimental conditions (Table 1). Three dose groups were subsequently established: a low-dose group (LE, 31.2 μg/mL), a medium-dose group (ME, 61.5 μg/mL), and a high-dose group (HE, 125 μg/mL), corresponding to 25%, 50%, and 100% of the MTC, respectively.

**Figure 5:**
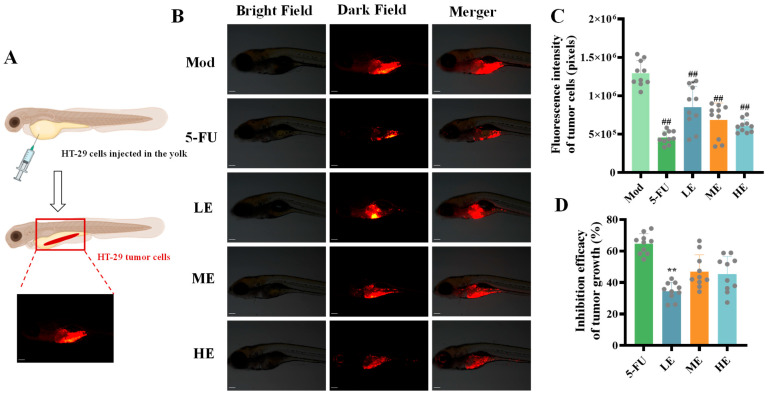
Anti-proliferative activity of ERPP was assessed in HT-29 zebrafish xenografts. Schematic diagram (**A**), fluorescence map of tumor cells and their quantification (**B**,**C**) and inhibition of tumor growth rate (**D**). Values are mean ± SE (*n* = 10). ^##^ *p* < 0.01 compared to Mod group, ** *p* < 0.01 compared to 5-FU group.

The tumor volume was quantitatively assessed via microscopy by analyzing the fluorescence intensity of HT-29 tumor cells (Figure 5A). Notably, xenografts treated with ERPP demonstrated significantly reduced tumor areas compared to those in the Mod group (Figure 5B,C). This result suggested that ERPP may suppress tumor growth by regulating cell proliferation. Specifically, when examining the inhibition rate of tumor growth, significant differences compared to 5-FU were observed only in the LE group, while no significant differences were noted between the ME and HE groups (Figure 5D). These results collectively demonstrated that high-dose ERPP exhibited antitumor efficacy equivalent to 5-FU in suppressing tumor progression in vivo.

#### 2.2.2. Effects of ERPP on Tumor Metastasis in HT-29 Zebrafish Xenografts

**Figure 6:**
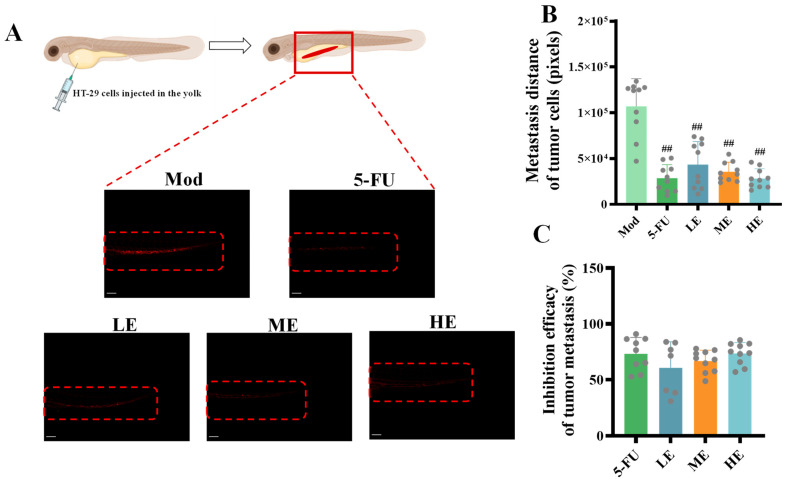
Anti-metastasis activity of ERPP was assessed in HT-29 zebrafish xenografts. Schematic diagram and fluorescence map of tumor cells (**A**), fluorescence quantification of tumor cells (**B**) and inhibition of tumor metastasis rate (**C**). Values are mean ± SE (*n* = 10). ^##^ *p* < 0.01 compared to Mod group.

The effects of ERPP on HT-29 cell migration were further investigated in vivo using a zebrafish xenograft model. Fluorescence imaging indicated significantly reduced tumor cell migration rates and attenuated fluorescence intensity in both the ERPP and 5-FU groups (Figure 6A,B). These results are consistent with the findings of our previous in vitro cell experiment, which demonstrated their tumor growth inhibitory effects. Quantitative analysis demonstrated no statistically significant difference between ERPP and 5-FU in suppressing tumor cell migration (Figure 6C). These results collectively indicated that ERPP effectively inhibited HT-29 cell migration in vivo.

#### 2.2.3. Effects of ERPP Treatments on Tumor Angiogenesis

**Figure 7:**
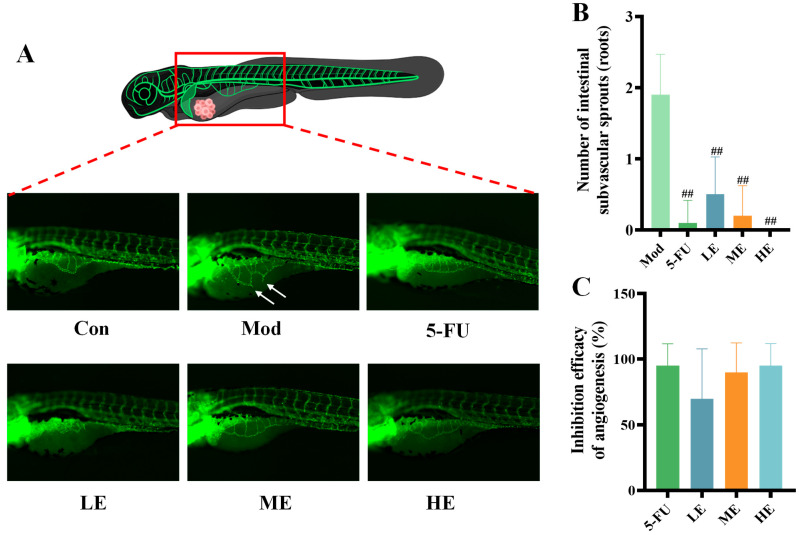
Antitumor angiogenesis activity of ERPP was assessed in HT-29 zebrafish xenografts. Fluorescence map of angiogenesis and their quantification (**A**,**B**) and inhibition of angiogenesis rate (**C**). Values are mean ± SE (*n* = 10). ^##^ *p* < 0.01 compared to Mod group.

The vasculature in Fil-1 transgenic xenografts was non-invasively visualized through fluorescent imaging (Figure 7A), with analysis specifically focused on tumor-proximal blood vessels critical for neoplastic maintenance and expansion. ERPP-treated zebrafish exhibited significantly attenuated tumor-associated angiogenesis (Figure 7B), particularly evidenced by the reduced fluorescence intensity of the subintestinal vessel plexus-derived vasculature compared to the Mod group. No significant differences were observed between the 5-FU and ERPP groups in angiogenic suppression efficacy (Figure 7C).

#### 2.2.4. Effects of ERPP Treatments on Cell Apoptosis in HT-29 Zebrafish Xenografts

**Figure 8:**
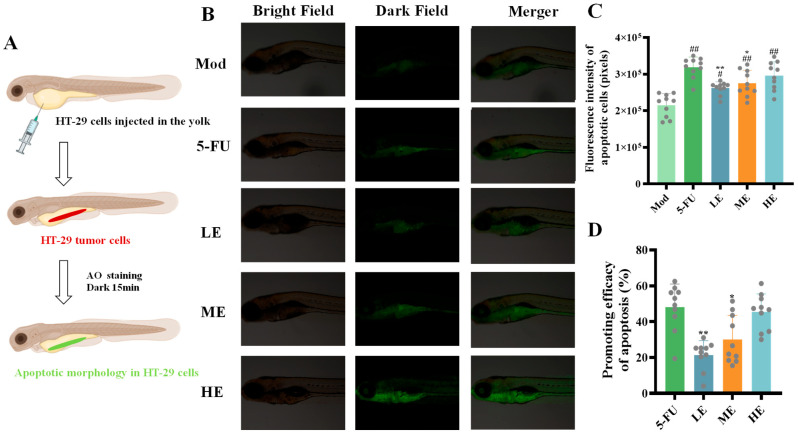
Antitumor angiogenesis activity of ERPP was assessed in HT-29 zebrafish xenografts. Schematic diagram (**A**), fluorescence map of tumor cells and their quantification (**B**,**C**) and promoting effect of apoptosis rate (**D**). Values are mean ± SE (n = 10). ^##^ *p* < 0.01, ^#^ *p* < 0.05 compared to Mod group, ** *p* < 0.01, * *p* < 0.05 compared to 5-FU group.

The cell studies above have demonstrated the tumor growth and metastasis inhibitory properties of ERPP, along with its ability to decrease tumor-associated angiogenesis. Subsequent investigations were conducted to evaluate its apoptosis-inducing efficacy in vivo. After 15 min of acridine orange (AO) staining under light-protected conditions, apoptotic cells were visualized as green fluorescence under microscopy (Figure 8A). A marked increase in green fluorescence intensity was observed in both the ERPP and 5-FU groups, indicating that both ERPP and 5-FU significantly promoted tumor cell apoptosis (Figure 8B,C). A quantitative analysis of pro-apoptotic rates revealed no statistically significant difference between high-dose ERPP and 5-FU (Figure 8D). These findings collectively demonstrated the pro-apoptotic effects of ERPP both in vitro and in vivo.

### 2.3. Research on Antitumor Mechanism of ERPP

**Figure 9:**
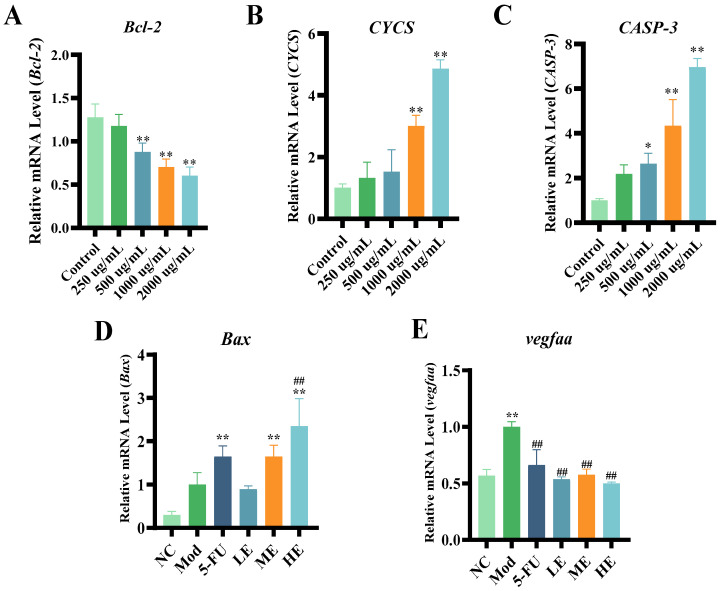
Effect of ERPP on mitochondrial apoptosis pathway. Expression of *Bcl-2* (**A**), *CYCS* (**B**), and *CASP-3* (**C**) gene. ** *p* < 0.01, * *p* < 0.05 compared to control group. Expression of *Bax* (**D**) and *vegfaa* (**E**) gene. Values are mean ± SE (*n* = 3). ** *p* < 0.01 compared to NC group, ^##^ *p* < 0.01 compared to Mod group.

Previous studies have demonstrated that polysaccharides, such as guava seed polysaccharides (GSF3), can cause the apoptosis of MCF-7 breast cancer cells by increasing the Bax/Bcl-2 ratio [28]. Additionally, Grifola frondosa polysaccharide (cGFP) has been shown to induce mitochondrial apoptosis in HepG_2_ cells via Bax upregulation and Bcl-2 downregulation [29]. To confirm its role in cancer cell apoptosis, the pro-apoptotic gene *Bax*, anti-apoptotic gene *Bcl-2*, *CYCS* gene, and *CASP-3* gene were assessed using RT-qPCR. ERPP treatment was found to significantly downregulate the anti-apoptotic gene *Bcl-2* and upregulate pro-apoptotic *Bax*. Moreover, it led to an increased expression of cytochrome C and *CASP-3* (*p* < 0.01) (Figure 9A–D). These findings align with the reported mechanisms of polysaccharide-induced tumor suppression, suggesting that ERPP may exert its antitumor effects through activating the endogenous mitochondrial apoptotic pathway.

Vascular endothelial growth factor A (*Vegfaa*) has been recognized to enhance the proliferation and migration of vascular endothelial cells [30]. Elevated expression levels of *vegfaa* in cancer have been widely documented [31], where it promotes tumor-associated neovascularization through microenvironmental remodeling, subsequently driving malignant progression and metastatic dissemination. In comparison with the Mod group, a significant downregulation of *vegfaa* expression was observed in the ERPP group, demonstrating a dose-dependent suppression of angiogenic signaling (Figure 9E). These findings suggested that the ERPP-mediated attenuation of peritumoral vascular angiogenesis contributed to tumor growth inhibition and metastasis prevention. These observations were consistent with the studies above demonstrating the antiangiogenic mechanisms of bioactive polysaccharides.

### 2.4. Antioxidant Activities of ERPP

#### 2.4.1. ABTS^•+^, DPPH•, and Hydroxyl Free Radical Scavenging Rate Analysis

**Figure 10:**
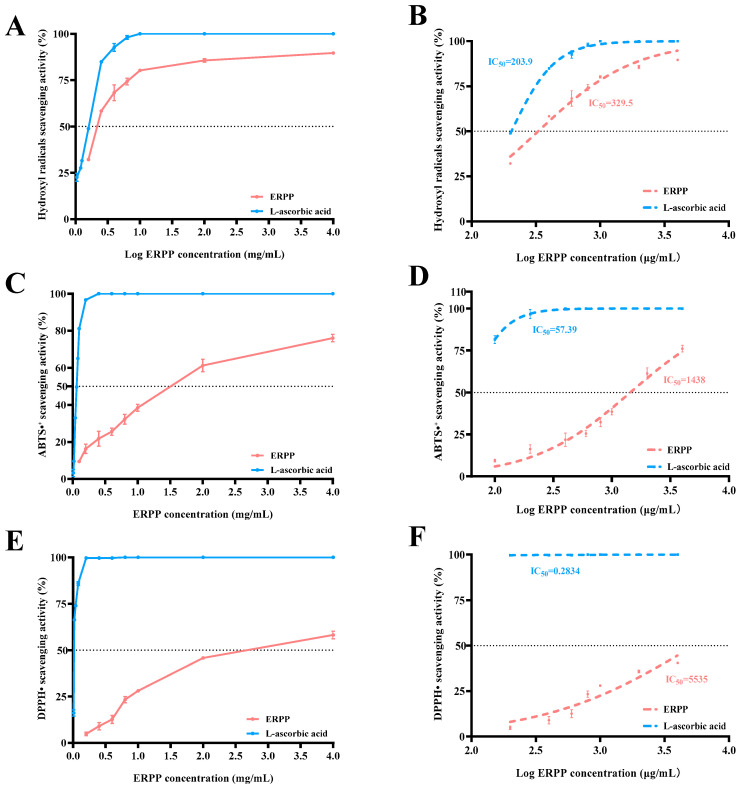
Antioxidant capacity of ERPP at different reaction concentrations on hydroxyl radical scavenging activity and IC_50_ (**A**,**B**), ABTS^•+^ scavenging activity and IC_50_ (**C**,**D**), and DPPH• scavenging activity and IC_50_ (**E**,**F**). Values are mean ± SE (*n* = 3).

Hydroxyl radicals have been reported to readily traverse cellular membranes, inducing tissue and cellular damage via interactions with biomolecules such as lipids, proteins, and DNA [32]. Consequently, effectively neutralizing hydroxyl radicals is critical for mitigating oxidative stress-related pathologies. As shown in Figure 10A, ERPP exhibited concentration-dependent hydroxyl radical scavenging activity, achieving an 80.2% radical scavenging rate at 4 mg/mL. This efficacy was comparable to L-ascorbic acid, a well-characterized antioxidant [33]. Dose–response analysis revealed parallel scavenging kinetics between ERPP and the positive control, confirming ERPP’s potent hydroxyl radical scavenging capacity.

The ABTS^•+^ method measures the total antioxidant capacity [34], while DPPH• radicals are relatively stable free radicals with a single electron [35]. As illustrated in Figure 10C,E, a concentration-dependent increase in ABTS^•+^ and DPPH• radical scavenging activity was observed for ERPP. At the maximal tested concentration (4 mg/mL), ERPP achieved scavenging rates of 75.04 ± 1.78% and 58.53 ± 1.09% for ABTS^•+^ and DPPH• radicals, respectively. In contrast, its hydroxyl radical scavenging capacity was significantly lower compared to these two radical species under identical experimental conditions.

Previous studies have established that polysaccharides reduce intracellular reactive oxygen species levels through direct hydroxyl radical neutralization, thereby inhibiting oxidative stress-mediated DNA damage and subsequent carcinogenesis [36]. For instance, ulvan, a sulfated polysaccharide isolated from *Ulva* species (*Ulvaceae* family), has been reported to exert anticancer and anti-inflammatory effects via antioxidant pathway modulation [37]. As shown in Figure 10B,D,F, the IC_50_ values for ERPP were found to be 329.5, 1438, and 5535 μg/mL for hydroxyl radical scavenging, ABTS^•+^ radical scavenging, and DPPH• radical scavenging, respectively. In summary, ERPP was demonstrated to exhibit potent antioxidant activity, particularly in hydroxyl radical scavenging.

#### 2.4.2. Effects of ERPP on Intracellular ROS

**Figure 11:**
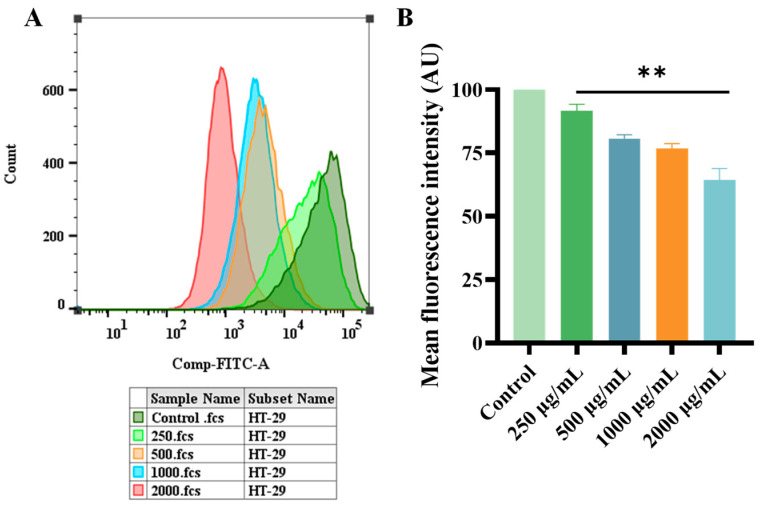
Effect of ERPP on ROS of HT-29 and its MFI value (**A**,**B**). Values are mean ± SE (*n* = 3). ** *p* < 0.01 compared to control group.

Reactive oxygen species (ROS), characterized by their transient nature and potent reactivity, are derived intracellularly from molecular oxygen [38]. Their impact on both tumorigenic cells and immune function is critically dependent on concentration. Our previous data above demonstrate that ERPP possesses significant hydroxyl radical scavenging capacity. Analysis revealed a dose-responsive attenuation of ROS mean fluorescence intensity (MFI) in HT-29 cells cultured under these conditions (Figure 11A,B). Experimental data demonstrate that ERPP treatment significantly attenuates intracellular ROS levels in tumor cells, and these findings exhibit significant alignment with prior published results [39].

## 3. Discussion

Polysaccharides have been recognized for their diverse pharmacological activities and broad therapeutic potential as versatile natural compounds [40]. Previous investigations have primarily emphasized the inhibitory effects of polysaccharides on cancer cell proliferation, such as the documented induction of apoptosis in A549 cells by *sea cucumber* polysaccharides through their antitumor properties [41]. This study was focused on exploring the antitumor mechanisms and antioxidant capacity of *R. philippinarum* polysaccharide (ERPP) by enzymatic hydrolysis. It was demonstrated that ERPP enhanced the anticancer responses by activating the endogenous mitochondrial apoptosis pathways. Additionally, ERPP was found to exhibit quantifiable antioxidant activity, which synergistically enhanced its tumor-suppressive effects. Collectively, these data indicated that ERPP could represent a potential functional food component or adjuvant therapy for colorectal cancer management.

Apoptosis has been recognized as a critical pathway for tumor cell death regulation in response to cytotoxic agents [42]. The *oyster*-derived polysaccharide OP-1 was demonstrated to suppress HepG-2 cell proliferation in a time-dependent manner through apoptosis induction [11]. *Starfish* polysaccharides have also been documented to inhibit the metastasis of HT-29 colorectal cancer cells [43]. In the present investigation, ERPP was observed to effectively inhibit tumor cell proliferation both in vitro HT-29 cultures and in zebrafish HT-29 xenograft models. Furthermore, ERPP-treated cells showed reduced migratory capacity and suppressed clonogenic potential. Mechanistic analysis revealed that the antitumor effects of ERPP were mediated through apoptosis induction, which is consistent with prior findings in related polysaccharide research.

The intrinsic apoptotic pathway, a classical regulatory cascade predominantly controlled by the Bcl-2 family, has been shown to be activated under external stimuli. Reduced Bcl-2 expression compromised mitochondrial integrity, resulting in cytochrome C over-release and apoptotic body formation, which subsequently triggered caspase family activation and apoptotic progression [44]. It has been demonstrated by studies that the expression of Caspase-3, Caspase-8, Caspase-9, and Bax was upregulated by the polysaccharide fucoidan, while the expression of Bcl-2 and CDK-2 was downregulated [45]. Fucoidan exerts antitumor effects primarily by inhibiting tumor cell viability, proliferation, and the metastatic dissemination of cancer cells from primary tumor sites to distant secondary sites [46]. Zhang R.J. et al. reported that *abalone* polysaccharide (AVP) induced dose-dependent apoptosis in MGC-803 cells by downregulating Bcl-2 and VEGF expression and upregulating Bax and p53 levels [12]. Similarly, the clam-derived F2.1 polysaccharide was found to modulate apoptosis-related proteins, such as by upregulating cytochrome C, cleaved caspase-3, and caspase-9 while downregulating Bcl-2, ultimately resulting in apoptotic cell death [47]. In the current study, we observed a similar mechanistic phenomenon, wherein ERPP mediated tumor cell apoptosis by activating the endogenous mitochondrial apoptosis pathway. This finding aligns with the established molecular mechanisms of polysaccharide-induced cytotoxicity reported in previous studies. However, further studies could be needed to explore how ERPP enters the cell.

Tumor angiogenesis is a well-known hallmark of malignant progression. Tumor-associated vasculature serves three critical functions: nutrient and oxygen supply for proliferation, metastatic dissemination facilitation, and immune evasion through endothelial-mediated immunomodulation [48]. Angiogenesis inhibition was demonstrated to induce tumor cell dormancy/apoptosis via hypoxia–nutrient deprivation while also reducing the metastatic potential through the suppression of circulatory system infiltration. In this investigation, ERPP significantly inhibited tumor angiogenesis, with an efficacy comparable to that of the positive control 5-FU. This could be one of the mechanisms through which ERPP exerts its antitumor effects in vivo.

Potent antioxidant capacity has been widely documented in marine-derived polysaccharides [38], including ERPP, with particular efficacy observed in hydroxyl radical scavenging. Hydroxyl radicals, as reactive oxygen species (ROS) constituents, are generated during cellular oxidative processes. The therapeutic depletion of these radicals was demonstrated to attenuate ROS accumulation within tumor microenvironments, consequently suppressing oncogenic signaling pathways.

## 4. Materials and Methods

### 4.1. Materials and Reagents

ERPP, provided by Qingdao Marine Functional Food and Health R&D Laboratory (Qingdao, China), was a polysaccharide extracted from *R. philippinarum* with a total sugar content of 91.41 ± 0.72% detected by the phenol–sulfuric acid method. All cellular models were obtained from the Cell Resource Center of the Shanghai Institutes for Biological Sciences, Chinese Academy of Sciences (Shanghai, China). DMEM/high-glucose culture medium was procured from HyClone (Logan, UT, USA). McCoy’s 5A and RPMI-1640 media were sourced from Procell Life Science & Technology Co., Ltd. (Wuhan, China). FBS was obtained from Thermo Fisher Scientific’s Gibco division (Grand Island, NY, USA). The Annexin V-FITC/PI apoptosis detection system, CCK-8 proliferation assay reagents, and JC-1 mitochondrial membrane potential detection kits were provided by Beyotime Institute of Biotechnology (Shanghai, China). All remaining chemical compounds met analytical-grade purity standards.

### 4.2. Sample Preparation of ERPP

*R. philippinarum* specimens were procured from a local market in Qingdao, Shandong Province, China. The clams were homogenized with distilled water (1:2, *w*:*v*) followed by enzymatic hydrolysis using trypsin (Deebio Pharmaceutical Co., Ltd., Chengdu, China) at 37 °C for 3 h. The hydrolysate was then centrifuged at 8000× *g* for 15 min, and the supernatant was fractionated through a 30 kDa molecular weight cut-off ultrafiltration membrane. Ethanol precipitation (4:1, *v*:*v*) was performed on the retentate, with subsequent filtration and lyophilization yielding *R. philippinarum* polysaccharides (ERPP).

The homogeneity of ERPP was confirmed by High-performance gel permeation chromatography (HPGPC), demonstrating a narrow and symmetrical polysaccharide peak at an elution time of 30.732 min (Figure S1A). The calibration equation derived from multiple molecular mass carbohydrates was as follows: Log Mw = −0.1761x + 11.0299 (R^2^ = 0.9927). The approximate molecular weight of ERPP was 414.949 kDa. Analysis by the PMP-HPLC method revealed that glucose was the primary monosaccharide component of ERPP (Figure S1B), compared to standard monosaccharides.

### 4.3. Cell Culture

A549 (human non-small cell lung cancer cells), B16 (mouse melanoma cells), HuH-7 (human hepatocellular carcinoma cells), MGC-803 (human gastric cancer cells), CT-26 (mouse colon cancer cells), and HT-29 (human colorectal carcinoma cells) were cultured in high-glucose Dulbecco’s Modified Eagle Medium (DMEM), RPMI-1640, or McCoy’s 5A medium, supplemented with 10% fetal bovine serum (FBS) and 1% penicillin–streptomycin. All cells were maintained in a humidified incubator at 37 °C with 5% CO_2_.

#### 4.3.1. Cell Counting Kit-8 Assay

Multiple cell lines (A549, B16, HuH-7, MGC-803, CT-26, and HT-29) were seeded into 96-well plates at a density of 5 × 10^5^ cells per well and cultured for 24 h. Subsequently, the cells were treated either with DMEM (control) or with varying concentrations of ERPP (200, 500, 800, 1000, 1500, and 2000 µg/mL) for an additional 48 h. Then, 10 μL of Cell Counting Kit-8 solution (CCK-8) was added to each well, followed by incubation at 37 °C for 0.5~1 h in the dark. Optical density (OD) was measured at 450 nm using a microplate reader. Cell viability (%) was calculated as follows:

Cell viability (%) = [(OD_3_ − OD_1_)/(OD_2_ − OD_1_)] × 100 (1)

where OD_1_, OD_2_, and OD_3_ represent the absorbance values of the blank (DMEM), control (DMEM and cells), and ERPP-treated groups (ERPP, DMEM and cells), respectively.

#### 4.3.2. Colony Formation

To assess the inhibitory effects of ERPP on HT-29 cell proliferation, cells were seeded into 6-well plates at 5 × 10^4^ cells/well and treated with ERPP (0, 250, 500, 1000, and 2000 μg/mL) for 24 h, then reseeded with 5 × 10^2^/well into a 6-well plate. The medium was refreshed with McCoy’s 5A every 72 h until the 8th day. The cells were then fixed with 4% paraformaldehyde, stained with 0.5% crystal violet for 10 min, and washed with PBS. Colony formation was imaged using an Amersham Imager AI680RGB (GE Healthcare, Hino, Japan) and quantified with Fiji Image J (V2.7.0).

#### 4.3.3. Wound Healing Assay

HT-29 cells were seeded into 6-well plates (2 × 10^5^ cells/well) in complete medium with 10% FBS. Following overnight culture, a linear wound was generated in the cell monolayer using a sterile 200 μL pipette tip. The cells were then treated with ERPP for 12, 24, or 48 h. Wound closure was imaged by inverted microscopy, and the migrated area was analyzed using Image J. The migration rate was calculated as follows:

Migration rate (%) = [(A_0_ − A_t_)/A_0_] × 100 (2)

where A_0_ and A_t_ represent the initial and post-migration wound areas, respectively.

#### 4.3.4. Cell Apoptosis

The induction of apoptosis by ERPP was analyzed via Annexin V-FITC/PI dual staining. The quadrants in the flow cytometric analysis were defined as follows: Q1 (Annexin V−/PI+, necrotic cells), Q2 (Annexin V+/PI+, late apoptotic cells), Q3 (Annexin V−/PI−, viable cells), and Q4 (Annexin V+/PI−, early apoptotic cells) [49]. Quantification was performed based on the percentage of cells in each quadrant, with each quadrant representing a distinct type of cell. HT-29 cells were seeded in 6-well plates at a density of 5 × 10^5^ cells/mL and treated with ERPP (250, 500, 1000, and 2000 μg/mL) and 5-fluorouracil (5-FU, positive control) for 48 h. The cells were harvested, incubated with 5 μL Annexin V-FITC and PI in the dark for 20 min, and then analyzed using a BD FACS Celesta flow cytometer (Becton Dickinson, Franklin Lakes, NJ, USA).

#### 4.3.5. Detection of Mitochondrial Membrane Potential (MMP)

The mitochondrial membrane potential (MMP) was evaluated using a JC-1 fluorescent probe. Briefly, the pretreated HT-29 cells were resuspended in 0.5 mL PBS and incubated with JC-1 staining solution at 37 °C for 20 min according to the manufacturer’s protocol (Mitochondrial Membrane Potential Assay Kit, Beyotime, Shanghai, China). Fluorescence intensity was quantified via flow cytometry (BD FACSCanto II, San Jose, CA, USA). For morphological validation, the identically pretreated cells were analyzed using an inverted fluorescence microscope (Axio Vert.A1, Carl Zeiss, Jena, Germany).

### 4.4. Animal Experiment

The wild-type AB strain and Fli-1 transgenic zebrafish lines were used for the different analyses. The zebrafish Fli-1 promoter is able to drive the expression of enhanced green fluorescent protein (EGFP) in all blood vessels during the entire embryogenesis process. The zebrafish was maintained according to standard procedure. Embryos were obtained from natural spawning and raised at 28 °C in fish water. The water quality parameters were set at 200 mg of instant sea salt per liter of reverse osmosis water, with a conductivity ranging from 450 to 550 μS/cm, a pH value between 6.5 and 8.5, and a hardness between 50 and 100 mg/L CaCO_3_. The fish were sourced from Huante Biological company’s fish breeding center under the experimental animal use permit number SYXK (Zhe) 2022-0004, and the breeding procedures adhered to the international AAALAC certification standards (Certification number: 001458). The age of embryos is indicated as days post fertilization (dpf) for all experimental data shown.

#### 4.4.1. Fluorescent Labeling of HT-29 Cells

For xenotransplantation, HT-29 cells were washed twice with phosphate-buffered saline (PBS), trypsinized, and resuspended in DMEM containing 5 μM CellTracker^TM^ CM-DiI Red Fluorescent dye (Thermo Fisher Scientific, Waltham, MA, USA). After incubation at 37 °C for 30 min, the cells were centrifuged at 1200× *g* for 5 min, washed twice with PBS to remove excess dye, and resuspended in PBS at a density of 1 × 10^6^ cells/mL for subsequent zebrafish yolk microinjection.

#### 4.4.2. The Establishment of a Zebrafish Tumor Transplantation Model

Wild-type AB strain and Fli-1 transgenic zebrafish embryos at 2 dpf were injected with approximately 200–250 DiI-labeled HT-29 cells into the inferior section of the yolk sac. After injection, a zebrafish tumor transplantation model was successfully established. The zebrafish model was then incubated at 35 °C until 3 dpf. Zebrafish displaying a consistent engraftment of tumor cells were selected at 3 dpf through microscopic examination and distributed randomly into 6-well plates, with 30 embryos per well. Water-soluble samples were administered, and 5-FU (100 ng/mL) was used as the positive control. Simultaneously, a model control group was established.

#### 4.4.3. Tumor Growth, Angiogenesis, and Metastatic Potential Quantification

Following a 48 h incubation at 35 °C, 10 zebrafish were randomly chosen from each experimental group for imaging under a fluorescence microscope (AZ100, Nikon, Yokohama, Japan). Images of both the wild-type AB strain and Fli-1 transgenic zebrafish were analyzed using NIS-Elements D 3.20 software to determine tumor size based on the area of each tumor (red fluorescent structure). A standardized area was selected for all samples to assess the impact of therapeutic agents on metastatic proliferation. The Maximum Testing Concentration (MTC) was determined by measuring the mortality of zebrafish in each experimental group. This allowed for the determination of the MTC of the samples on the zebrafish model.

#### 4.4.4. Tumor Cells Apoptosis

After 48 h of incubation at 35 °C, all experimental groups were subjected to acridine orange (AO) staining for 15 min under no-light conditions. After three washes with standard dilution buffer, 10 zebrafish for each group were randomly selected and imaged under a fluorescence microscope (Nikon Eclipse Ti2, Yokohama, Japan). Fluorescence data were acquired using NIS-Elements D software (version 3.20), and apoptotic cell fluorescence intensity was quantified. The pro-apoptotic efficacy of the test compounds was statistically evaluated based on this parameter.

Apoptosis Rate (%) = (Sample − Mod)/Mod × 100%(3)

### 4.5. Quantitative Real-Time Reverse Transcription Polymerase Chain Reaction (RT-qPCR)

According to previous research [17], HT-29 cells were pretreated following the protocol described above. Total RNA was isolated using an RNA Quick Extraction Kit (BioTeke, Wuxi, China). Quantitative reverse transcription PCRs (qRT-PCRs) were prepared with ChamQ Universal SYBR Green qPCR Master Mix (Vazyme, Nanjing, China), gene-specific primers, and cDNA templates in a 20 μL reaction volume. Amplification was performed on a CFX Connect Real-Time PCR Detection System (BioRad, Hercules, CA, USA) under standardized thermal cycling conditions. Primer sequences are listed in Table 2. Target gene expression levels were normalized to the endogenous control β-actin and calculated via the 2^−ΔΔCt^ method.

### 4.6. Antioxidant Activity Analysis

#### 4.6.1. ABTS^•+^, DPPH•, and Hydroxyl Free Radical Scavenging Rate Analysis

ERPP solutions were prepared at gradient concentrations (0.25–4 mg/mL) through serial dilution. L-ascorbic acid (0.01–4 mg/mL) was utilized as the positive control in antioxidant capacity evaluations, including ABTS^•+^, hydroxyl radical, and DPPH• scavenging rate assays. The half-maximal inhibitory concentration (IC_50_), defined as the concentration of the sample required to achieve 50% radical scavenging, was determined using linear regression analysis based on the relationship between the percentage of scavenging and the concentration of ERPP. IC_50_ values were calculated by GraphPad Prism8.2.1 software based on the nonlinear fitting method.

As reported in previous research [17], the ABTS^•+^, hydroxyl radical, and DPPH• scavenging rate assays of ERPP were evaluated, using L-ascorbic acid as the reference standard. The hydroxyl radical scavenging rate assay was conducted with the Nanjing Jiancheng Kit (Nanjing, China).

#### 4.6.2. Intracellular Reactive Oxygen Species (ROS) Detection

Consistent with a prior methodology [17], HT-29 cells underwent identical pretreatment. After triple PBS washing and supernatant removal, cells were resuspended in DCFH-DA-containing medium (10 μM) and incubated at 37 °C for 20 min under dark conditions. Subsequently, washed cells were resuspended in 500 μL PBS, with the quantification of intracellular ROS levels using DCF fluorescence intensity via flow cytometry.

### 4.7. Statistical Analysis

All results were expressed as the mean ± SE, and a one-way analysis of variance (ANOVA) with a post hoc Tukey test was used to identify significant differences among each group. Values of *p* < 0.05 and *p* < 0.01 were considered to indicate statistically significant and extreme significant differences, respectively.

## 5. Conclusions

**Figure 12:**
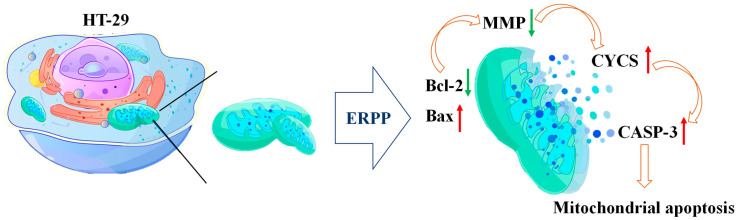
Possible antitumor mechanism of ERPP in cell apoptosis with detailed pathways.

ERPP was demonstrated to induce apoptosis in HT-29 colorectal cancer cells through both in vitro and in vivo zebrafish xenograft models. Mechanistic investigations via RT-qPCR revealed that ERPP-mediated apoptosis was associated with the downregulation of the anti-apoptotic gene *Bcl-2* and the upregulation of the pro-apoptotic gene *Bax*, resulting in MMP destabilization. JC-1 fluorescence assays confirmed MMP collapse in ERPP-treated cells, which triggered cytochrome C (*CYCS*) release into the cytosol. The released cytochrome C bound to cytoplasmic dATP, forming apoptosomes that activated the executioner Caspase-3 (*CASP-3*), thereby initiating the intrinsic mitochondrial apoptotic pathway (Figure 12). Additionally, ERPP exhibited potent antioxidant activity, particularly in hydroxyl radical scavenging, as evidenced by dose-dependent radical inhibition assays. These findings collectively demonstrated that ERPP, as a marine-derived nature polysaccharide, promotes tumor cell apoptosis via mitochondrial dysfunction and redox modulation. These findings provide a mechanistic basis for the future application of ERPP as a nutraceutical agent or adjunctive therapy in colorectal carcinoma treatment.

## Figures and Tables

**Table 1:** Experimental results of ERPP on MCT in zebrafish model (*n* = 30).

Concentration (μg/mL)	Number of Zebrafish Fatalities	Mortality Rate (%)	Phenotype of Zebrafish
15.6	0	0	Consistent with the model control group
31.2	0	0	
61.5	0	0	
125	0	0	
250	16	63	—

**Table 2:** Primer sequences of apoptotic genes.

Gene	Primer Sequence
*Bcl-2*	F: ATCGCCCTGTGGATGACTGAGT R: GCCAGGAGAAATCAAACAGAGGC
*CASP-3*	F: AGAGGGGATCGTTGTAGAAGTC R: ACAGTCCAGTTCTGTACCACG
*CYCS*	F: CTTTGGGCGGAAGACAGGTC R: TTATTGGCGGCTGTGTAAGAG
*Bax*	F: GACTTGGGAGCTGCACTTCT R: TCCGATCTGCTGCAAACACT
*vegfaa*	F: TCCCGACAGAGACACGAAAC R: CATCTTGGCTTTTCACATCTTTCT
*β-actin*	F: TCGAGCAGGAGATGGGAACC R: CTCGTGGATACCGCAAGATTC

## Data Availability

The original contributions presented in the study are included in the article and Supplementary Materials, further inquiries can be directed to the corresponding author.

## Supplementary Materials

The following supporting information can be downloaded at https://www.mdpi.com/article/10.3390/md23080304/s1, Figure S1: The molecular weight and monosaccharide composition of ERPP. (A): HPGPC of ERPP. (B) PMP-HPLC of ERPP.
