# Supplementary material for: Antitumor Activity of Ruditapes philippinarum Polysaccharides Through Mitochondrial Apoptosis in Cellular and Zebrafish Models
Source: Mar Drugs. 2025 Jul 29;23(8):304. doi: 10.3390/md23080304 (PMC12387552; doi:10.3390/md23080304)
Supplement: Supplementary file 1 [file marinedrugs-23-00304-s001.zip › marinedrugs-3738919-supplementary.pdf]

Supplementary Materials

Antitumor Activity of *Ruditapes philippinarum* Polysaccharides Through Mitochondrial Apoptosis in Cellular and Zebrafish Models

Mengyue Liu [1], Weixia Wang [2], Haoran Wang [3], Shuang Zhao [1], Dongli Yin [2], Haijun Zhang [2], Chunze Zou [4], Shengcan Zou [2], Jia Yu [1,*] and Yuxi Wei [1,*]

[1] College of Life Sciences, Qingdao University, Qingdao 266071, China; lmy02112021@163.com (M.L.); zhaoshuang2544@163.com (S.Z.)
[2] Qingdao Yihai Industry Holdings Co., Ltd., Qingdao 266105, China; wangweixia@chenland.cn (W.W.); yindongli@chenland.cn (D.Y.); zhanghaijun@chenland.cn (H.Z.); zoushengcan@chenland.cn (S.Z.)
[3] College of Materials Science and Engineering, Qingdao University, Qingdao 266071, China; 19817801579@163.com
[4] Donald Bren School of Information and Computer Sciences, University of California, Irvine, CA 92697, USA; chunzez@uci.edu
* Correspondence: yujiaqdu@163.com (J.Y.); yuxiw729@163.com (Y.W.); Tel.: +86-53285953227 (J.Y.); +86-13356886682 (Y.W.)

Monosaccharide composition analysis

The molecular weight of ERPP was detected by the HPGPC method. In brief, each dextran standards with different relative molecular weights and the ERPP were accurately weighed to 5 mg, dissolved in 1mL of 0.05 M NaCl solution to obtain 5 mg/mL solutions, and then centrifuged for 10 min at 8,000 rpm. The supernatant was filtered and transferred to a 2 mL injection vial. The purity and the molecular weight of the ERPP were analyzed using a Waters differential refractive index detector and three Water-soluble SEC (GFC) polymer matrix columns (8 × 300 mm) in series. The column temperature was maintained at 40 ℃, the flow rate was maintained at 0.65 mL/min, and the injection volume was set to 30 μL. Monosaccharide standards (rhamnose, arabinose, galactose, glucose, xylose, mannose, glucuronic acid, galacturonic acid, glucosamine hydrochloride, and amino galactose hydrochloride monosaccharides) were accurately weighed to 5.0 mg each. After dissolution, the volume was adjusted to 10mL using a volumetric flask, and the solutions were diluted to 10, 20, 40, 60, 80, 100, 200, 500 μg/mL (fucose was diluted to 20, 40, 60, 80, 100, 200, 500, 1000 μg/mL). Standard curves were established by preparing standard samples of different concentrations. The samples were then treated with TFA, heated, and dried under nitrogen. Subsequently, sodium hydroxide solution and PMP methanol solution were added. After vortex mixing, the reaction was carried out in a water bath at 70 ℃ for 1 h. The mixture was extracted with chloroform, which was discarded, and the remaining aqueous phase was adjusted to a final volume of 1 mL with water. To detect the monosaccharide composition of ERPP, a Thermo U 3000 liquid chromatography system (Thermo, USA) equipped with ZORBAX Eclipse XDB-C18 was used. The system operated with a flow rate of 0.8 mL/min, a column temperature of 30 ℃, a detection wavelength of 250 nm, an injection volume of 10 μL.

The homogeneity of ERPP were confirmed by HPGPC demonstrating a narrow and symmetrical polysaccharide peak at an elution time of 30.732 min (Figure S1 A). The calibration equation derived from multiple molecular mass carbohydrates was: Log Mw= -0.1761x + 11.0299 (R^2= 0.9927). The approximate molecular weight of ERPP were 414.949 kDa. Analysis by the PMP-HPLC method revealed that glucose was the primary

monosaccharide component of the ERPP (Figure S1 B), compared to standard monosaccharides.

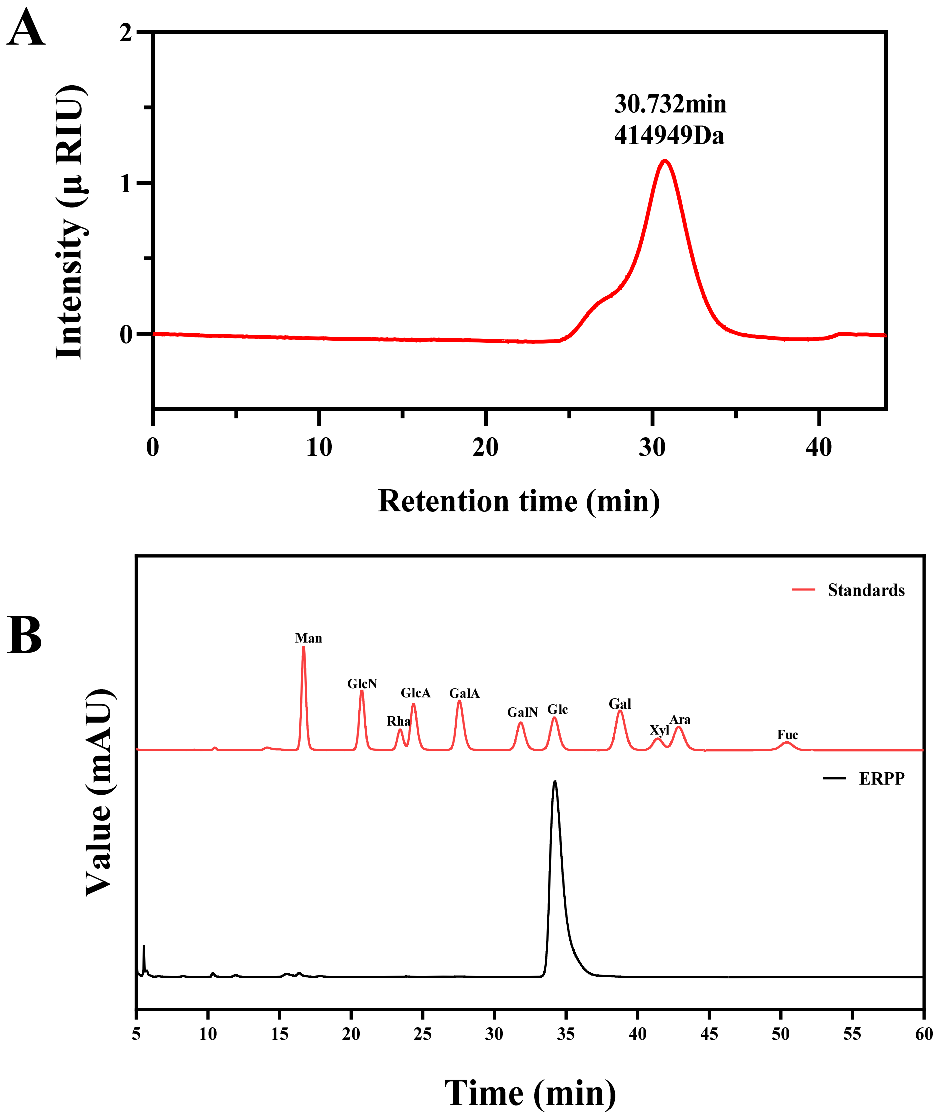

Figure S1: The molecular weight and monosaccharide composition of ERPP. (A): HPGPC of ERPP. (B) PMP-HPLC of ERPP.
